# Solitary Plasmacytoma of the Jugular Foramen: A Case Report and Review of the Differential Diagnosis

**DOI:** 10.7759/cureus.35592

**Published:** 2023-02-28

**Authors:** Fred Hsu

**Affiliations:** 1 Radiation Oncology, BC Cancer–Abbotsford Centre, Abbotsford, CAN

**Keywords:** multiple myeloma, radiotherapy, paraganglioma, plasmacytoma, jugular foramen

## Abstract

Jugular foramen tumours are uncommon, deeply located, and eloquently situated, making their diagnosis and management challenging. Paragangliomas and other benign tumours comprise the large majority of lesions in this region, but malignant tumours are occasionally identified. We report a unique case of a solitary plasmacytoma of the jugular foramen resembling a jugulotympanic paraganglioma. A solitary plasmacytoma of the jugular foramen is both rare in location and in disease presentation, as most plasma cell neoplasms are diagnosed as multiple myeloma. Our 75-year-old patient presented with symptoms typical for a jugular foramen tumour. Although there are radiographic features which help differentiate paragangliomas from other benign and malignant tumours, plasmacytomas are highly vascular and can demonstrate a local infiltrative spread which can mimic the radiographic appearance of a paraganglioma. Clinicians should consider plasma cell neoplasms in the differential when faced with an unusual presentation of a jugular foramen lesion. Our patient was treated with definitive radiotherapy to 45 Gy, which was very effective local treatment for the solitary plasmacytoma.

## Introduction

The jugular foramen is situated on the floor of the posterior fossa posterolateral to the carotid canal between the occipital bone and petrous temporal bone. Tumours in this region are uncommon and can arise from structures within the foramen or from adjacent tissues. The large majority of jugular foramen tumours are benign and include paragangliomas, neural sheath tumours, and meningiomas. Paragangliomas, also known as glomus jugulare, represent the most common lesion of the jugular foramen, constituting 81% of tumours in one study [1]. These tumours originate from paraganglia or glomus cells found in the adventitia of the jugular bulb, bony walls of the tympanic canals, or in the bone of the promontory [2]. Paragangliomas are highly vascular and, although histologically benign, can demonstrate extensive bone, vascular, and cranial nerve invasion. Patients with jugular foramen paragangliomas typically present with hearing loss and tinnitus, which can progress to vertigo, dysphagia, and lower cranial neuropathies following tumour growth.

Schwannomas and meningiomas constitute the majority of non-paraganglioma tumours of the jugular foramen. Jugular foramen meningiomas, like paragangliomas, can demonstrate a locally invasive growth pattern with extensive bone infiltration. These tumours can develop from the leptomeninges intrinsic to the jugular foramen in the jugular bulb or extrinsically in the posterior fossa with extension into the jugular foramen [2]. In comparison, schwannomas are also histologically benign but non-infiltrative. Schwannomas of the jugular foramen usually arise from the glossopharyngeal nerve or vagus nerve [1].

Malignant tumours of the jugular foramen are much less frequent, although the incidence is not well reported given the broad differential in this group. In most cases, malignant tumours arise from adjacent tissues and include chordomas, chondrosarcomas, and hemangiopericytomas. Metastases can also occur in the jugular foramen, usually from lung, breast, or prostate primary malignancies [2]. While plasma cell neoplasms involving the jugular foramen have been sparsely reported in literature, this finding has been in the context of multiple myeloma and have not been solitary. With institutional ethics approval, we present a rare case of a patient with a solitary plasmacytoma of the jugular foramen resembling a jugulotympanic paraganglioma.

## Case presentation

A 75-year-old Caucasian male presented to hospital with acute onset dizziness and a six-month history of right-sided hearing loss. He described recent right shoulder pain and difficulty raising his right arm above 90 degrees at the shoulder. He denied speech, swallowing, and visual symptoms. On assessment his gait was ataxic and drifted toward the left. His mobility and functional status were significantly limited (Karnofsky Performance Status 60). On neurologic examination, he had weakness abducting his right arm at the shoulder. On otoscopic examination the external auditory canals and tympanic membranes were normal in appearance. His medical history was only significant for type II diabetes and a myocardial infarction two years prior. He had no history of solid organ or hematologic malignancies.

**Figure 1:**
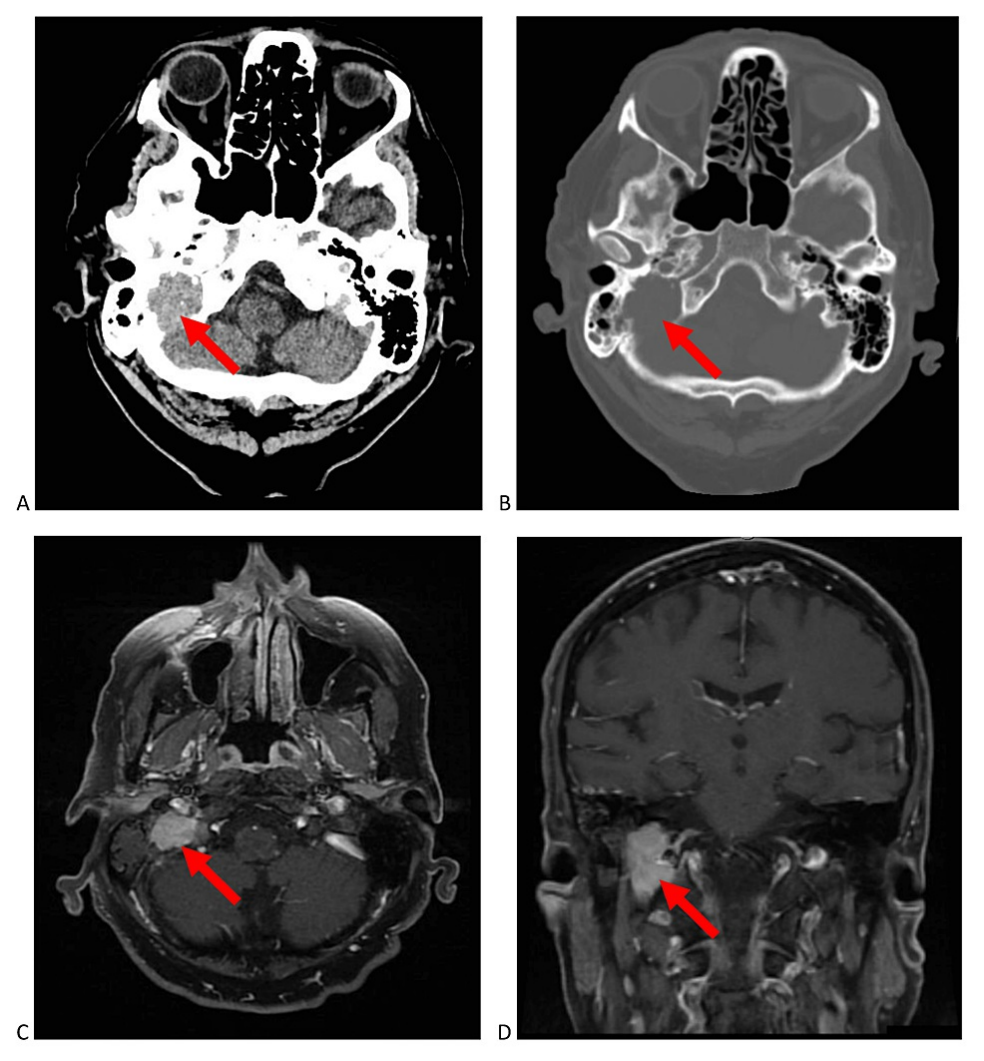
Brain imaging of the tumour arising in the right jugular foramen at presentation. (A) soft tissue axial non-contrast computed tomography (CT) and (B) bone-window axial non-contrast CT showing an expansile mass in the right jugular foramen causing osteolysis and “moth-eaten” borders of the adjacent petrous bone. (C) axial and (D) coronal T1-weighted post-contrast magnetic resonance imaging (MRI) showing marked uniform enhancement of the right jugular foramen mass. The superior extent of the tumour is just inferior to the vestibular apparatus.

A computed tomography (CT) scan of the head showed a heterogeneously hyperdense mass centered within the right jugular foramen and involving the right temporal bone measuring 2.2 x 2.0 x 2.8 cm. There was significant osteolysis and an irregular “moth-eaten” appearance. On magnetic resonance imaging (MRI), the lesion in the jugular fossa extended superiorly to the level of the cochlea, with no direct involvement of the cochlea or vestibular apparatus. The lesion was contiguous with the carotid canal, but there was no evidence of direct involvement of the carotid artery or middle ear space. The lesion was isointense on pre-contrast T1-weighted sequences, hyperintense on T2 sequences, and showed uniform contrast enhancement with gadolinium. A “salt-and-pepper” type appearance was not demonstrated. The CT and MRI at presentation is shown in Figure 1.

The clinical presentation in combination with the tumour location, disease pattern, and signal characteristics on imaging were consistent with a paraganglioma, which is the most common type of tumour in this location. The differential diagnosis included meningioma, schwannoma, and neurofibroma. As such, a biopsy was deferred as it was felt to be somewhat challenging and the patient was managed under this presumptive diagnosis. The lesion was not felt to be amenable to complete surgical excision. When considering the patients older age and poor functional status, the patient was recommended radiation therapy. As paragangliomas are typically benign and slow-growing, there was no immediate indication for expedited treatment. In combination with institutional delays, the patient waited three months from the time of his first presentation for further management, during which his right shoulder symptoms, ataxia, and hearing loss acutely worsened.

**Figure 2:**
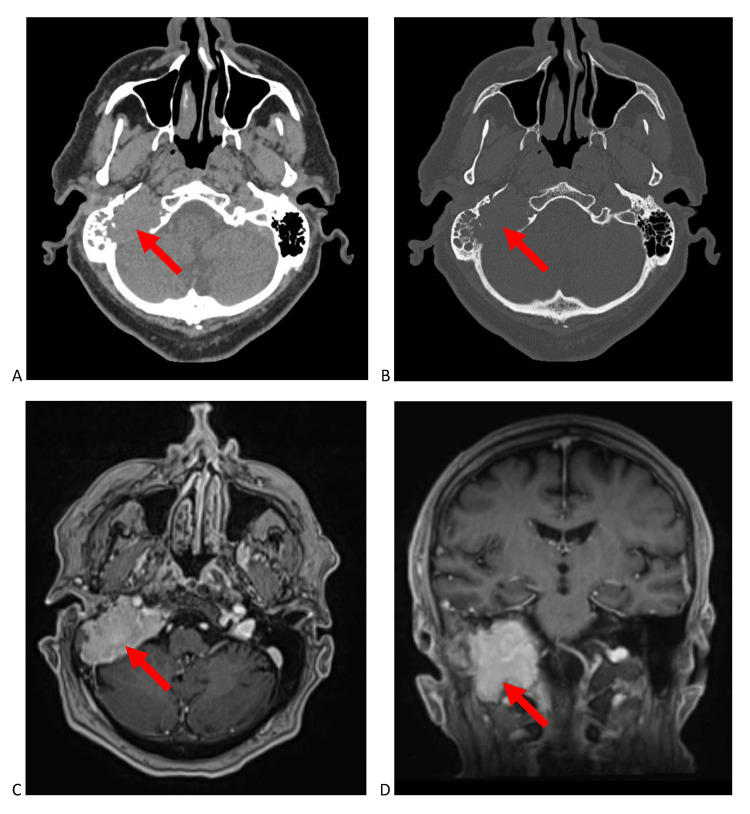
Brain imaging of the tumour in the right jugular foramen at three months in the absence of treatment. (A) soft tissue axial non-contrast computed tomography (CT) and (B) bone-window axial non-contrast CT showing marked interval enlargement of the tumour and further osteolysis. (C) axial and (D) coronal T1-weighted post-contrast magnetic resonance imaging (MRI) showing further extension into the mastoid air spaces and extension to the external auditory canal with obliteration of the vestibular apparatus.

A repeat CT at 10 weeks and MRI at 12 weeks after initial presentation showed marked interval enlargement of the right jugular foramen lesion, now measuring 4.0 x 2.0 x 4.1 cm (Figure 2). There was new osteolysis of the posterior wall of the external auditory canal with extension of soft tissue into the canal and involvement of the tympanic membrane. The rapid growth raised concern for a malignant process. A CT scan of the chest, abdomen, and pelvis at 12 weeks showed no evidence of an alternate primary malignancy or metastases elsewhere.

**Figure 3:**
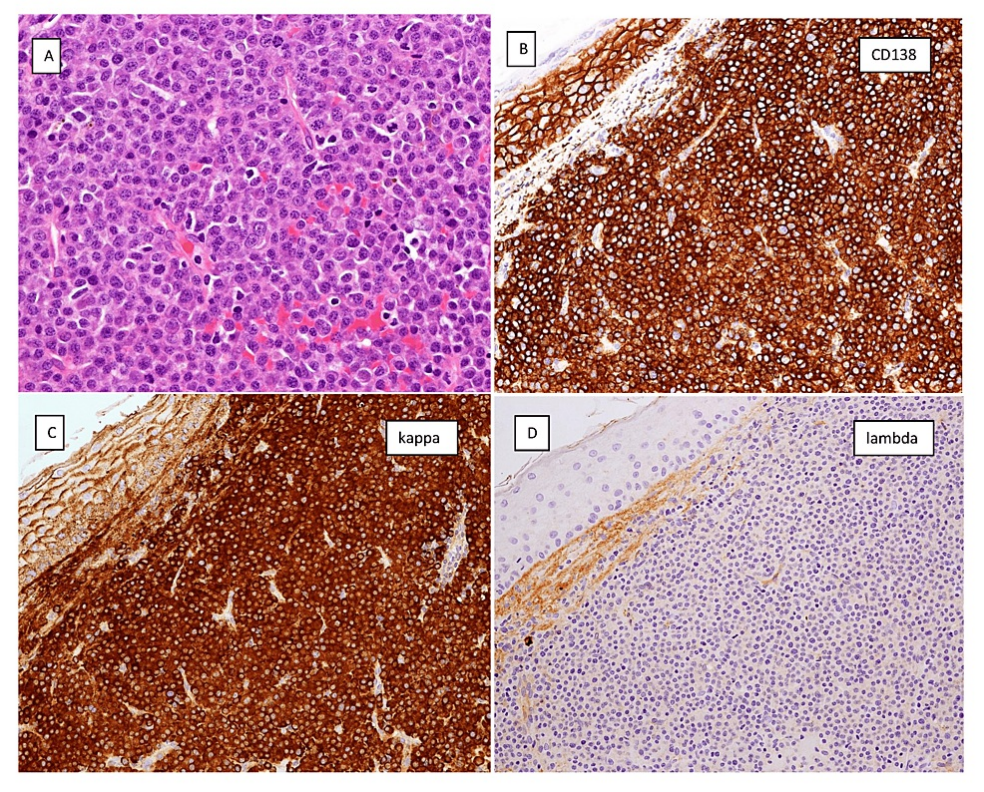
Pathology images from right external ear canal biopsy. (A) Hematoxylin and eosin stained sections of the tumour at 400x magnification showing infiltration of soft tissue by a large aggregate of lymphoid cells consistent with an atypical plasma cell proliferation. (B) Immunohistochemical studies reveal the atypical plasma cells are CD138 positive. (C-D) In situ hybridization studies show reactivity to kappa light chain and minimal to absent reactivity to lambda light chain.

The patient had a repeat otoscopic examination at 12 weeks now showing a pulsatile mass in the inferior middle ear space and a red mass in the external auditory canal. Biopsies of the mass from the external auditory canal described a highly vascularized tumour. The pathology was interpreted as a plasma cell neoplasm. Microscopically, there was an atypical plasma cell proliferation with an increased nuclear-cytoplasmic ratio and occasional nucleoli. Small lymphoid cells were very rare. By immunohistochemistry the malignant plasma cells were positive for CD138 and showed kappa light chain restriction. A subset of these cells expressed CD20. The atypical cells were negative for CD3, CD45, CD79a, EMA, S100, synaptophysin, pancytokeratin, CD99, and STAT6. Ki-67 proliferation rate was moderate at 30%. The pathology is presented in Figure 3.

**Figure 4:**
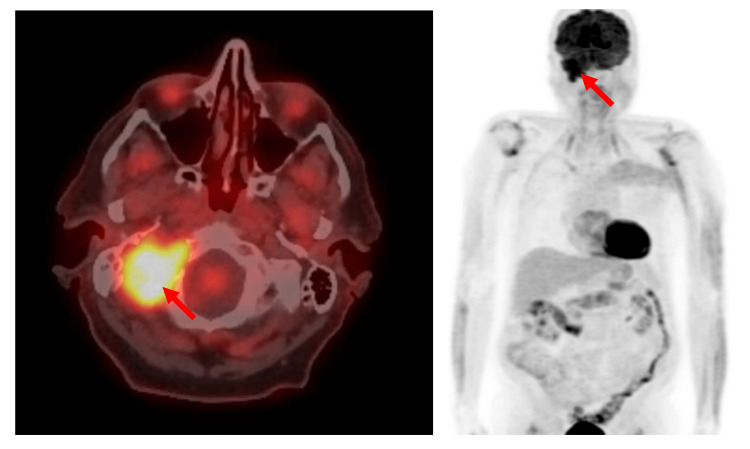
Positron emission tomography (PET) staging at baseline. Left hand side: axial image of the head displayed in “hot-iron” showing intense metabolic activity of the right jugular foramen plasmacytoma. Right hand side: coronal image of the body showing the solitary right jugular foramen plasmacytoma in the absence of radiographic evidence of systemic disease.

A complete blood count at 14 weeks showed all values within normal range: hemoglobin 143 g/L, white blood cell 7.0 x 10^9^/L, mean corpuscular volume 95 fL, and platelets 259 x 10^9^/L. On peripheral blood smear review, there were no rouleaux formation of red cells, while neutrophils showed toxic changes. Serum biochemistry showed normal serum calcium 2.36 mmol/L, normal electrolytes, normal lactate dehydrogenase 118 U/L, and normal creatinine 78 µmol/L. Beta-2 microglobulin was normal at 1.9 mg/L. Serum protein electrophoresis reported a monoclonal band (3.5 g/L) in the slow gamma region with no associated suppression of the normal gamma globulins. Immunofixation identifies the discrete band as immunoglobulin(Ig)G kappa paraprotein. Electrophoresis of urine (24-hour urine) reports a trace, non-quantifiable discrete band detected in the gamma region with immunofixation demonstrating a faintly visible discrete band as free kappa light chain paraprotein. A bone marrow aspirate and trephine biopsy reported no evidence of a plasma cell disorder. Staging with 18-fluoro-deoxyglucose (FDG) positron emission tomography (PET) imaging demonstrated intense uptake associated with the right jugular foramen lesion with no other FDG-avid or destructive bone lesions (Figure 4). Specifically, there were no findings to suggest multiple myeloma. The patient’s diagnosis was consistent with “a plasmacytoma with associated monoclonal gammopathy of undetermined significance”.

**Figure 5:**
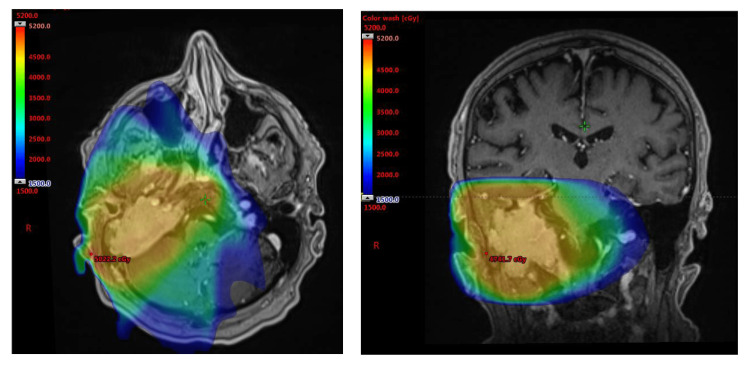
Isodose curves for definitive radiation therapy Isodose curves for definitive radiation therapy using an intensity-modulated radiotherapy technique covering the visible tumour volume without intentional coverage of the regional lymph nodes. Total dose of 45 Gy in 25 fractions.

The patient’s solitary plasmacytoma was treated with definitive radiotherapy to a total dose of 45 Gy in 25 fractions. Radiotherapy was given using an intensity-modulated radiotherapy technique. The primary tumour was delineated as the gross tumour volume on the planning CT scan with the assistance of fused T1-weighted MRI imaging. There was a 1 cm margin expansion to create a clinical target volume which was increased to 2 cm along bone and reduced to 0.5 cm within the adjacent brain and brainstem. In the absence of visible nodal involvement there was no prophylactic inclusion of lymph nodes along the head and neck region. An additional margin expansion of 0.5 cm was used for a planning target volume (PTV). The radiation dose was prescribed to the PTV. The radiotherapy treatment plan showing the isodose curves is presented in Figure 5.

**Figure 6:**
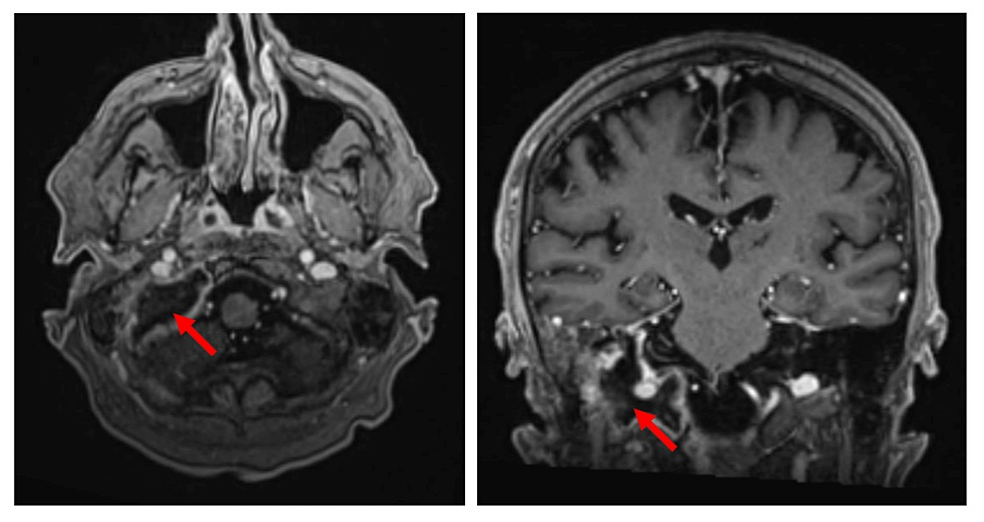
Brain imaging two months after radiotherapy treatment of the jugular foramen plasmacytoma. Axial (left hand side image) and coronal (right hand side image) T1-weighted post-contrast magnetic resonance imaging (MRI) showing complete cystic degeneration of the tumour which is no longer contrast enhancing.

**Figure 7:**
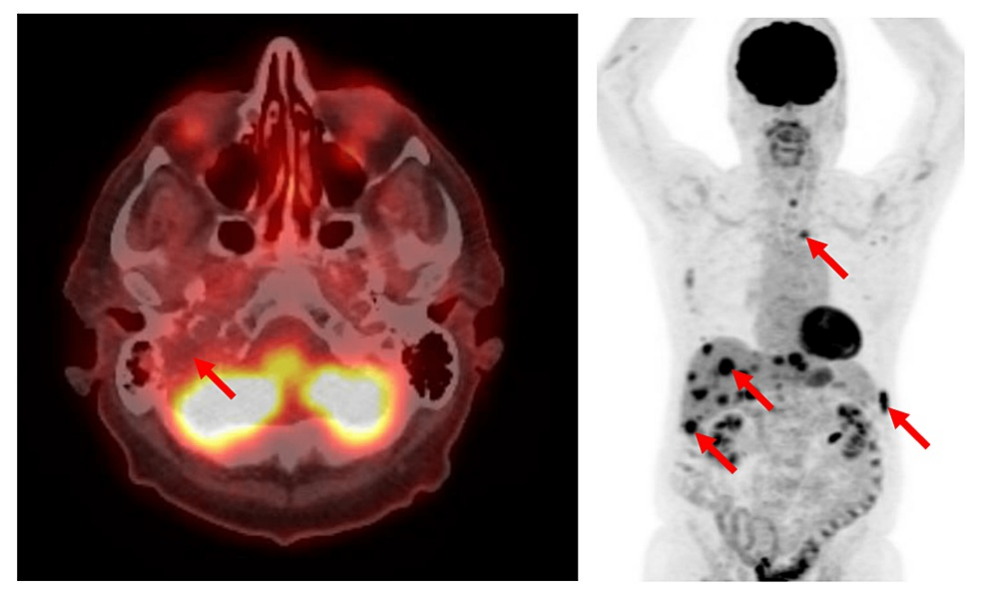
Positron emission tomography (PET) 10 months after completion of radiotherapy for the right jugular foramen plasmacytoma. Left hand side: axial image of the head displayed in “hot-iron” showing that the treated tumour is no longer metabolically active after 45 Gy of radiotherapy. Right hand side: coronal image of the body showing multiple new liver and bone metastases.

A post-treatment MRI two months after radiotherapy completion showed complete cystic degeneration of the mass with no contrast enhancement in keeping with a complete local response (Figure 6). Unfortunately, 10 months after radiotherapy completion, the patient developed multiple FDG-avid metastases involving the liver and new mildly FDG-avid radiolucent bone lesions on PET (Figure 7). This was confirmed with a liver biopsy showing plasma cell dyscrasia and excess kappa light chain expression. For multiple myeloma the patient was recommended a two-drug regimen of weekly subcutaneous bortezomib calculated as 1.3 mg/m^2^ body surface area and weekly dexamethasone 20 mg when considering his frailty. However, his weakness, fatigue, and immobility progressed, and he died two months later from aspiration pneumonia complicated with septicemia.

## Discussion

The diagnosis of jugular foramen tumours is often done after clinical examination and radiologic assessment [1]. Paragangliomas tend to erode bone while its spread follows the path of least resistance, including mastoid air cell tracts, vascular channels, and neural foramina [2]. On CT imaging, this pattern of spread produces a characteristic “moth-eaten” pattern of destruction in the temporal bone. This is different than neural sheath tumours, such as schwannomas and neurofibromas, which are non-infiltrative and demonstrate smoothly scalloped, well-corticated enlargement of the jugular foramen [3]. This is also different than meningiomas which typically demonstrate hyperostosis and sclerotic changes in addition to bone erosion [4]. Paragangliomas characteristically demonstrate intense contrast-enhancement on MRI indicative of its hypervascularity. Additionally, on T2-weighted MRI, a “salt-and-pepper” appearance is distinctive, related to the high-flow vascular voids within these vascular tumours [5,6]. Our patient had tumour infiltration following vascular channels with extensive infiltration of the mastoid air spaces and bone erosion, giving a “moth-eaten” appearance on CT similar to the bone destruction associated with paragangliomas. Further, the very vascular plasmacytoma showed intense contrast enhancement on MRI, allowing the lesion to mimic a paraganglioma. However, the classic “salt-and-pepper” appearance was absent on T2-weighted sequences. Most important to our case was the marked interval increase in size of the lesion over three months, which is suggestive of a malignant process rather than that of a benign paraganglioma, neural sheath tumour, or meningioma. Jugular foramen paragangliomas are rarely malignant, with malignant transformation identified by lymph node or distant metastatic spread [7]. Metastases in the jugular foramen can also present with contrast enhancement on T1-weighted MRI and an osteolytic destructive appearance similar to this case. However, in our patient, the jugular foramen lesion was solitary with no evidence of a primary malignancy elsewhere or other metastases on CT or PET imaging during staging. Additionally, metastases usually lack hyperintensity on T2-weighted MRI or have flow voids unless they are very vascular [2].

Malignant primary tumours involving the jugular foramen are uncommon and usually arise from adjacent tissues. The differential includes gliomas, hemangiopericytomas, hemangioblastomas, lymphomas, and hemangiomas. Some tumours can originate from the adjacent petrous bone or clivus and extend into the jugular foramen, such as chordomas, chondrosarcomas, fibrosarcomas, and epidermoid tumours. Other tumours can extend from the parapharyngeal space, such as rhabdomyosarcomas and nasopharyngeal carcinomas [2]. The diverse group of malignant primary tumours has varied presentations, some of which can have radiologic appearances similar to paragangliomas. Vascular tumours such as hemangiopericytomas and hemangiomas can share the same contrast enhancement signal characteristics on MRI as paragangliomas which are also very vascular. Chordomas and chondrosarcomas demonstrate irregular, infiltrative bone destruction. However, unlike paragangliomas, these tumours are typically peripherally enhancing and often contain calcifications [2]. Disease location and extension in relation to the jugular foramen may distinguish some tumours, such as gliomas, rhabdomyosarcomas, and nasopharyngeal carcinomas from paragangliomas. However, extrinsic lesions can sometimes mimic intrinsic lesions of the jugular foramen, emphasizing the importance of a biopsy, particularly when disease appearance or behaviour is atypical.

Plasma cell neoplasms consist of clonal plasma cells that are terminally differentiated B cells characterized by immunoglobulin secretion. The majority of plasma cell neoplasms are diagnosed as multiple myeloma. Only a small proportion of plasma cell neoplasms (5-6%) will manifest as a solitary plasmacytoma, either in bone or in extramedullary tissues, making them uncommon [8]. As such, a solitary plasmacytoma of the jugular foramen is exceedingly unusual both in location and disease presentation. The degree of bone destruction around the jugular foramen makes it difficult to determine whether the tumour originated in the bony skull base with extension into the jugular foramen or if the tumour was extramedullary with subsequent bony involvement. Solitary bone plasmacytomas are more common, frequently occur in the axial skeleton, and have a high risk of progression to multiple myeloma. Solitary extramedullary plasmacytomas are less common but occur mostly in the head and neck region, such as the nasal cavity, paranasal sinus, and nasopharynx [8].

In our literature search we found three case reports of plasma cell neoplasms involving the jugular foramen, all in patients with multiple myeloma [9-11]. How et al. [9] described a 29-year-old female who presented with hearing loss and a large tumour in the right jugular foramen with extensive osseous erosion and a “moth-eaten” appearance similar to our case. Their patient, however, had abnormal pre-operative bloodwork showing a disproportionate anemia and protein gap which led to a finding of IgA kappa monoclonal gammopathy. Their trans-mastoid biopsy of the jugular foramen lesion showed a plasma cell neoplasm, while a staging PET scan and bone marrow biopsy confirmed multiple myeloma. Similarly, Oushy et al. [10] reported on a 59-year-old female with cranial neuropathies secondary to a lytic lesion in the left jugular foramen. A biopsy of an adjacent occipital lesion identified a plasma cell neoplasm, while serum studies, skeletal survey, and bone marrow biopsy demonstrated multiple myeloma. Sokhi et al. [11] reported on a 57-year-old female being treated for multiple myeloma who subsequently developed cranial neuropathies secondary to progressive myeloma in the left base of skull extending to the jugular foramen. In our unique case there was no prior history or concurrent presentation with multiple myeloma. At presentation our patient did not demonstrate bloodwork abnormalities or end-organ damage associated with multiple myeloma such as anemia, hypercalcemia, or renal insufficiency. There was no PET-CT evidence of distant lesions and the bone marrow biopsy was absent for clonal plasma cells, meeting the diagnostic criteria for a solitary plasmacytoma as defined by the International Myeloma Working Group [12]. In our patient the primary treatment was definitive radiotherapy, unlike chemotherapy as the primary treatment for multiple myeloma as used in these other case reports.

Definitive surgical resection was not a viable treatment option for obtaining clear margins with acceptable morbidity in our patient. Further, it was unlikely that surgery would negate the need for radiotherapy in the postoperative period given the high risk of local recurrence. Plasma cell neoplasms are radiosensitive tumours and radiotherapy alone for solitary plasmacytomas provides excellent local control that may translate into a durable remission and even cure [13]. While the optimal dose of definitive radiotherapy for solitary plasmacytomas is not well established, the National Comprehensive Cancer Network recommends a dose of 40-50 Gy [8,13]. Our patient was treated using 45 Gy with a favorable local response. Unfortunately, long term local control could not be evaluated as he succumbed to disease related complications.

## Conclusions

While the large majority of jugular foramen tumours are attributed to paragangliomas and other benign lesions, an unusual disease behaviour should prompt clinicians to consider alternative diagnoses. Although uncommon, plasma cell neoplasms of the jugular foramen should be a consideration in patients with unusual presentations. Plasmacytomas are highly vascular and can demonstrate an infiltrative local spread that can mimic the radiographic appearance of a paraganglioma. In these situations, obtaining a pathologic diagnosis is particularly important for directing management.
